# Toluidine Blue for the Determination of Binding of Anionic Polysaccharides to Lipid Raft Domains by Absorption Spectroscopy

**DOI:** 10.3390/membranes15050139

**Published:** 2025-05-02

**Authors:** Sandra Gębczyńska, Julia Gdowska, Agata Mikos, Iga Gawrońska, Teresa Janas, Aleksander Czogalla, Tadeusz Janas

**Affiliations:** 1 Institute of Biology, University of Opole, Kominka 6, 45-032 Opole, Poland; 128919@student.uni.opole.pl (S.G.); julia.gdowska@uni.opole.pl (J.G.); 130994@student.uni.opole.pl (A.M.); 117941@student.uni.opole.pl (I.G.); teresa.janas@uni.opole.pl (T.J.); 2 Department of Cytobiochemistry, Faculty of Biotechnology, University of Wroclaw, F. Joliot-Curie 14a, 50-383 Wrocław, Poland; aleksander.czogalla@uwr.edu.pl

**Keywords:** absorption spectroscopy, liposomes, polysialic acid, polygalacturonic acid, lipid rafts, toluidine blue

## Abstract

The complexes of negatively charged polysaccharides with lipid vesicles have been shown to have applications in medicine, bioremediation, water purification, and construction of nano-biosensors. This article presents research on the formation of these complexes based on the interactions between three types of liposomes, DOPC liposomes (which contain a lipid bilayer in the liquid-disordered (Ld) state), RAFT liposomes (which contain liquid-ordered (Lo) lipid raft domains surrounded by lipids in the Ld state) and SPH–CHL liposomes (which contain a lipid bilayer in the Lo state), and two selected anionic polysaccharides, polysialic acid (PSA) and polygalacturonic acid (PGA). The analysis was conducted using a toluidine blue (TB) probe and the absorption spectroscopy technique. In contrast to DOPC and SPH–CHL liposomes, binding of negatively charged PSA or PGA chains to RAFT liposomes induced a TB absorption maximum shift from 630 nm to 560 nm. The obtained results indicate that toluidine blue can be applied for monitoring the formation of these nano-complexes, and that the boundaries between Ld/Lo domains within membranes in RAFT liposomes can significantly enhance the binding affinity of negatively charged polysaccharides to the lipid bilayer surface. The observed metachromatic shift in TB absorption suggests that negatively charged PSA and PGA chains interact with the Ld/Lo boundaries within RAFT liposome membranes.

## 1. Introduction

Polysaccharides interact with biological membranes in ways that have significant implications for their use in medicine and industry. Complexes of polysaccharides with lipid vesicles and extracellular vesicles have been explored for biomaterial development [1], extracellular vesicle isolation [2], and biosensor applications [3]. Additionally, these complexes are being evaluated for use in bioremediation [4,5,6] and water purification [7]. Due to the complexity of biological membranes, researchers often utilize lipid vesicles as models to better understand these interactions [8,9].

Polysialic acid (PSA) is a polyanionic polymer present on the surface of both exosomes and cancer cells [10,11,12]. It is a highly hydrated polyanion because its sialic acid (Sia) monomers carry a negative charge at physiological pH due to the presence of a carboxyl group. In humans, PSA consists of N-acetylneuraminic acid monomers arranged in a helical structure. Its function is linked to its ability to regulate repulsive and attractive interactions and modulate charge density and pH on membrane surfaces [13]. PSA chains are primarily associated with membranes, where they can be linked via glycosphingolipids, integral membrane glycoproteins, or glycosylphosphatidylinositol-anchored glycoproteins [10]. In eukaryotic cells, two polysialyltransferases involved in PSA biosynthesis are found in the Golgi complex [10]. Studies suggest that PSA plays a role as a multivalent bridge mediating interactions within the lipid bilayer, disrupting membrane aggregates, and facilitating *trans* interactions between adjacent membranes [13]. PSA has biomedical applications as a biomaterial [14] and in drug delivery systems designed to inhibit tumor growth [15,16,17]. Research has demonstrated that PSA-modified liposomes extend the circulation time of chemotherapeutic agents such as epirubicin [18] and enhance the efficacy of docetaxel in targeting tumor-associated macrophages [19]. Additionally, PSA-based micelles loaded with paclitaxel are being explored for cancer treatment [20]. PSA/protamine nanocapsules containing insulin have also been tested for their potential to reduce blood glucose levels in diabetic models [21].

Polygalacturonic acid (PGA) is a naturally occurring biopolymer in plant cells, composed of galacturonic acid residues linked by α-1,4 bonds. The molecular weight of PGA typically ranges between 25 and 100 kDa. It is a primary component of pectin and is commonly used in pharmaceutical formulations as an excipient. PGA and its salts are also utilized in anticancer therapies and as dietary supplements in the form of zinc, magnesium, and iron salts [22,23,24,25]. Degradation products of PGA, such as oligo-galacturonic acids, can influence bacterial flora and have been identified as key molecules in plant growth and development [24,25]. PGA is currently being studied for its potential in drug delivery applications [26,27], particularly in hepatocyte regeneration and controlled-release therapies [28].

Membrane rafts are dynamic microdomains within cellular membranes that are enriched with sphingolipids, cholesterol, and a subset of membrane proteins [29]. They are distinguished from other membrane regions by their unique composition and physical properties, allowing them to serve as platforms for multiprotein complexes and signaling pathways [30,31,32]. Lipid rafts contribute to cellular processes such as receptor-mediated signaling, immune responses, membrane trafficking, and RNA binding [29,32]. Due to their functional significance, lipid rafts have been proposed as therapeutic targets for diseases including neurodegeneration, angiogenesis, infections, and inflammatory conditions [33,34,35,36,37]. Studies on model membrane systems demonstrated that cholesterol facilitates phase separation in ternary mixtures with phospholipids and the formation of liquid-ordered (Lo) domains. The latter can be considered as equivalents of raft domains in cellular membranes.

This study examines how the coexistence of liquid-disordered (Ld) and liquid-ordered (Lo) lipid microdomains within the lipid bilayer, which resemble membrane raft formation (we use the name “lipid raft domains” for these microdomains), influences the binding of anionic polysaccharides to liposomal membranes using toluidine blue as a spectroscopic probe.

## 2. Materials and Methods

*Chemicals*

Lipids used for the preparation of DOPC, RAFT, and SPH–CHL liposomes, 1,2-dioleoyl-sn-glycero-3-phosphocholine (DOPC), N-stearoyl-D-erythro-sphingosylphosphorylcholine (stearoyl sphingomyelin, SPH), and cholesterol (CHL), were procured from Avanti Polar Lipids (Alabaster, AL, USA). Polysialic acid (PSA, poly-2,8-acetylneuraminic acid, sodium salt derived from *Escherichia coli* K1), polygalacturonic acid (PGA, sodium salt from citrus fruit), and toluidine blue (TB) were obtained from Sigma-Aldrich (St. Louis, MO, USA). Buffer A (50 mM HEPES, pH 7.0, 50 mM NaCl, 5 mM MgCl_2_, 2 mM CaCl_2_) was used in the experiments. TB, a metachromatic thiazine dye, demonstrates acidophilic properties and selectively stains acidic tissue components [38]. TB is a basic dye that is also used to determine the surface charge of cells [39]. It has the maximum absorption at 630 nm. The binding of TB to the negative charges present on the membrane surface results in a metachromatic shift in its maximum absorption to 560 nm [40].

*Preparation of Liposomes*

Liposomes (RAFT liposomes consisting of DOPC, SM, and CHL in the molar ratio of 6:3:1, plain DOPC liposomes, and SPH–CHL liposomes consisting of SM and CHL in the molar ratio of 6:4) were prepared following the extrusion method [41,42]. The purchased lipids were mixed in designated concentrations, evaporated under nitrogen gas, and subsequently rehydrated to a 20 mg/mL lipid concentration, using buffer A maintained at 65 °C. To produce unilamellar liposomes, the suspension underwent extrusion through a 100 nm membrane filter using an Avanti MiniExtruder (Avanti Polar Lipids, Alabaster, AL, USA).

*Absorption Spectroscopy*

Absorption spectra of toluidine blue in the range of 450–700 nm were recorded using a JASCO V-550 spectrophotometer (JASCO Corporation, Tokyo, Japan) at 21 °C. The absorbance values at wavelengths 560 nm (A_560_) and 630 nm (A_630_) were obtained both from the absorption spectra and single-wavelength measurements. The spectra were corrected for the background (light scattering) including the liposomes and the polysaccharides at the same concentrations but without the dye.

*Titration with Liposomes at Fixed Concentration of Toluidine Blue and PSA*

A mixture containing 150 µL of buffer A, 1.5 μL of a 100 mg/mL PSA aqueous solution (final concentration in the cuvette: 1 mg/mL), and 3 μL of a 500 μM toluidine blue aqueous solution (final concentration in the cuvette: 10 μM) was titrated with liposomes at final concentrations ranging from 0 to 0.4 mg/mL.

*Titration with PSA at a Constant Toluidine Blue and Liposome Concentration*

A solution comprising 150 µL of buffer A, 3.75 μL of liposomes at a 20 mg/mL concentration (final concentration in the cuvette: 0.5 mg/mL), and 3 μL of a 500 μM toluidine blue aqueous solution (final concentration in the cuvette: 10 μM) was titrated with PSA aqueous solutions at final concentrations ranging from 0 to 1 mg/mL. Absorption was measured at the wavelengths of 560 nm (A_560_) and 630 nm (A_630_) at 21 °C.

*Titration with Toluidine Blue at Fixed Liposome and PSA Concentrations*

A mixture containing 150 µL of buffer A, 3.75 μL of liposomes at a 20 mg/mL concentration (final concentration in the cuvette: 0.5 mg/mL), and 1.5 μL of a 100 mg/mL PSA aqueous solution (final concentration in the cuvette: 1 mg/mL) was titrated with toluidine blue at concentrations from 0 to 20.12 μM. Absorbance was recorded at the wavelengths of 560 nm (A_560_) and 630 nm (A_630_) at 21 °C.

*Titration with Toluidine Blue at Fixed Liposome and PGA Concentrations*

Liposomes at a final concentration of 0.5 mg/mL in the cuvette and PGA at a final concentration of 1.0 mg/mL were titrated with toluidine blue at concentrations ranging from 0 to 18.9 µM. Absorption was measured at the wavelengths of 560 nm (A_560_) and 630 nm (A_630_) at 21 °C.

*Titration with PGA at Fixed Toluidine Blue and Liposome Concentrations*

Liposomes at a final concentration of 0.4 mg/mL in the cuvette and a toluidine blue aqueous solution at a final concentration of 10 µM were titrated with PGA at concentrations from 0 to 1 mg/mL. Absorbance was measured at the wavelengths of 560 nm (A_560_) and 630 nm (A_630_) at 21 °C.

*Titration with Liposomes at Fixed Toluidine Blue and PGA Concentrations*

An aqueous toluidine blue solution at a final concentration of 10 µM in the cuvette and PGA at a final concentration of 1 mg/mL were titrated with liposomes at final concentrations ranging from 0 to 2.0 mg/mL. Absorption was recorded at the wavelengths of 560 nm (A_560_) and 630 nm (A_630_) at 21 °C.

*Parameters Used for Calculations*

The following parameters were used for absorption calculations: absorption ratio (R_1_ = A_560_/A_630_) and absorption difference ratio (R_2_ = (A_630_ − A_560_) (for samples with sequentially increasing PSA or PGA concentrations) / (A_630_ − A_560_) (for the sample with the lowest PSA or PGA concentration)).

## 3. Results

### 3.1. Binding of Polysialic Acid (PSA) to Liposomes Evaluated Using Toluidine Blue Absorption

#### 3.1.1. Titration with Toluidine Blue at Fixed Liposome and Polysialic Acid Concentrations

Supplementary Figure S1a (in the Supplementary Materials) presents the absorption spectra of toluidine blue (TB) at concentrations ranging from 0 to 20.12 µM, while maintaining constant concentrations of polysialic acid (PSA) at 1 mg/mL and RAFT liposomes at 0.5 mg/mL. As the TB concentration increased, absorption intensities at 560 nm and 630 nm also increased. The minimum TB concentration at which a notable absorbance increase was observed was 3.75 µM. For DOPC liposomes, the increase in absorbance at 630 nm was significantly more pronounced than at 560 nm (Supplementary Figure S1b).

**Figure 1:**
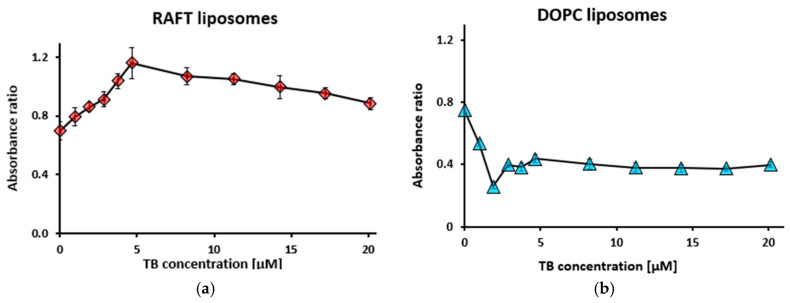
Absorbance ratio R_1_ = A_560_/A_630_ for RAFT liposomes (**a**) and DOPC liposomes (**b**) during titration with toluidine blue (TB). Polysialic acid concentration was 1 mg/mL, liposome concentration—0.5 mg/mL. Values are the means ± SE of 3 independent experiments.

From the absorbance data in Figure S1, absorbance ratio R_1_ for RAFT and DOPC liposomes was calculated (Figure 1).

The absorbance ratio (R_1_) for RAFT liposomes increased sharply, reaching 1.16 at a TB concentration of 4.66 µM, followed by a gradual decrease (Figure 1a). For DOPC liposomes, the lowest absorption ratio of 0.26 was recorded at 1.9 µM TB, stabilizing thereafter at the level of 0.4 (Figure 1b).

#### 3.1.2. Titration with Polysialic Acid at Fixed Toluidine Blue and Liposome Concentrations

In titrations with PSA concentrations ranging from 0 to 1 mg/mL, while maintaining constant TB levels at 10 µM and RAFT liposome concentrations at 0.4 mg/mL, the absorbance at 560 nm increased, whereas the absorbance at 630 nm decreased (Supplementary Figure S2). In the case of DOPC liposomes, absorbance decreased both at 560 nm and 630 nm; in the case of SPH–CHL liposomes, absorbance increased both at 560 nm and 630 nm.

**Figure 2:**
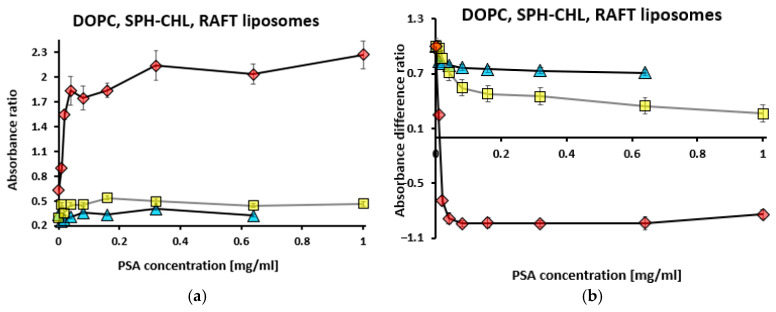
Absorbance ratio R_1_ = A_560_/A_630_ (**a**) for RAFT liposomes (red diamonds), DOPC liposomes (blue triangles) and SPH–CHL liposomes (yellow squares) along with absorbance difference ratio R_2_ (**b**) for RAFT, DOPC, and SPH–CHL liposomes during titration with polysialic acid (PSA). Toluidine blue concentration was 10 µM, liposome concentration—0.4 mg/mL. R_2_ = (A_630_ – A_560_) (for samples with sequentially increasing concentrations) / (A_630_ – A_560_) (for the sample with the lowest concentration). Values are the means ± SE of 3 independent experiments.

Using the absorbance data from Figure S2, both the absorbance ratio (R_1_) and the absorbance difference ratio (R_2_) were determined for RAFT, DOPC, and SPH–CHL liposomes (Figure 2).

For RAFT liposomes, the absorbance ratio (R_1_) increased sharply to 1.84 at 0.04 mg/mL PSA, followed by a gradual increase (Figure 2a). This suggests that the negative surface potential of the liposomes increased, indicating PSA attachment. In contrast, for DOPC and SPH–CHL liposomes, R_1_ increased to 0.36 at 0.08 mg/mL PSA and to 0.54 at 0.16 mg/mL PSA, respectively, before stabilizing. Therefore, RAFT liposomes exhibited a much higher R_1_ value compared to DOPC and SPH–CHL liposomes. The absorbance difference ratio (R_2_) for RAFT liposomes decreased sharply (Figure 2b) to −0.94 at 0.08 mg/mL PSA, followed by a slight increase. On the contrary, for DOPC and SPH–CHL liposomes, the value of R_2_ remained relatively stable at 0.7 and 1.0, respectively.

#### 3.1.3. Titration with Liposomes at Fixed Toluidine Blue and Polysialic Acid Concentrations

During titration with liposomes at concentrations ranging from 0 to 0.4 mg/mL, while keeping PSA constant at 1 mg/mL and TB at 10 µM, RAFT liposomes exhibited an increased absorbance at 560 nm and a decreased absorbance at 630 nm (Supplementary Figure S3a). The lowest liposome concentration at which PSA binding occurred was 0.2 mg/mL. In contrast, DOPC liposome titration resulted in an increased absorbance at both 560 nm and 630 nm (Supplementary Figure S3b).

**Figure 3:**
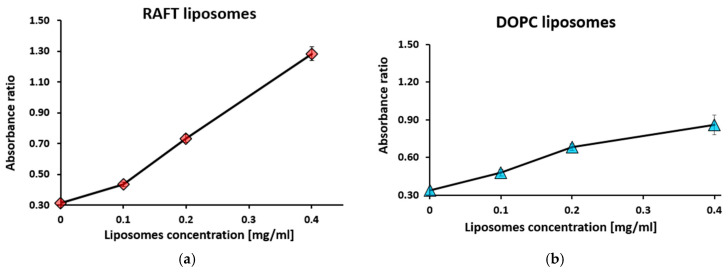
Absorbance ratio R_1_ = A_560_/A_630_ for RAFT liposomes (**a**) and DOPC liposomes (**b**) during titration with liposomes. Toluidine blue concentration was 10 µM, polysialic acid concentration—1 mg/mL. Values are the means ± SE of 3 independent experiments.

From the absorbance data in Figure S3, the absorbance ratio (R_1_) for RAFT and DOPC liposomes was determined (Figure 3).

For RAFT liposomes, absorbance ratio R_1_ increased with an increasing liposome concentration, reaching a peak of 1.28 at 0.4 mg/mL liposome concentration (Figure 3a). For DOPC liposome titration, the absorbance ratio increased with an increasing liposome concentration up to 0.86 at 0.4 mg/mL liposome concentration (Figure 3b).

### 3.2. Binding of Polygalacturonic Acid (PGA) to Liposomes Measured Using Toluidine Blue Absorption

#### 3.2.1. Titration with Toluidine Blue at Constant Liposome and Polygalacturonic Acid Concentrations

Supplementary Figure S4 displays the results of TB titration for concentrations ranging from 0 to 18.9 µM, maintaining constant RAFT liposome and DOPC liposome concentrations at 0.5 mg/mL, as well as a fixed polygalacturonic acid (PGA) concentration of 1 mg/mL. In the presence of RAFT liposomes, absorbance at both 560 nm and 630 nm increased as the TB concentration increased (Supplementary Figure S4a). For DOPC liposomes, absorbance increased substantially at 630 nm but not at 560 nm (Supplementary Figure S4b). The lowest TB concentration at which a significant absorbance increase was detected was 4.42 µM.

**Figure 4:**
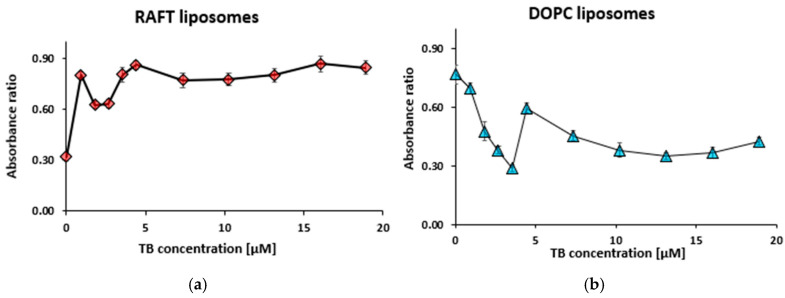
Absorbance ratio R_1_ = A_560_/A_630_ for RAFT liposomes (**a**) and DOPC liposomes (**b**) during titration with toluidine blue. Polygalacturonic acid concentration was 1 mg/mL, liposome concentration—0.5 mg/mL. Values are the means ± SE of 3 independent experiments.

From the absorbance data in Figure S4, absorbance ratio R_1_ for RAFT and DOPC liposomes was calculated (Figure 4).

The absorbance ratio graph for RAFT liposomes has two regions: first—an increase from 0.32 at 0 µM TB, reaching a peak of 0.86 at 4.42 µM TB; second—a plateau region (Figure 4a). The absorbance ratio graph for DOPC liposomes also displays two regions: first region—a sharp decrease from 0.77 at 0 µM TB to 0.29 at 3.56 μM TB; the second region—a slow decrease from 0.60 at 4.42 mM TB, with stabilization as the TB concentration increased (Figure 4b). TB titrations both in the presence of PGA (Figure 4) and in the presence of PSA (Figure 1) displayed similar two-region isotherms. A possible explanation for this phenomenon can come from an increase in the incorporation of TB molecules into the lipid bilayer with the increasing TB concentration in the buffer, followed by interactions between dye monomers and aggregation of TB molecules in the membrane.

#### 3.2.2. Titration with Polygalacturonic Acid at Constant Toluidine Blue and Liposome Concentrations

During titration with PGA at concentrations from 0 to 1.0 mg/mL, with a fixed RAFT liposome concentration of 0.4 mg/mL and constant TB at 10 µM, the absorbance at 560 nm increased, while the absorption at 630 nm decreased (Supplementary Figure S5a). The minimum PGA concentration required for observable binding to liposome membranes was 0.01 mg/mL. For DOPC liposomes (Supplementary Figure S5b) and SPH–CHL liposomes (Supplementary Figure S5c), increases in absorbance occurred at both 560 nm and 630 nm.

**Figure 5:**
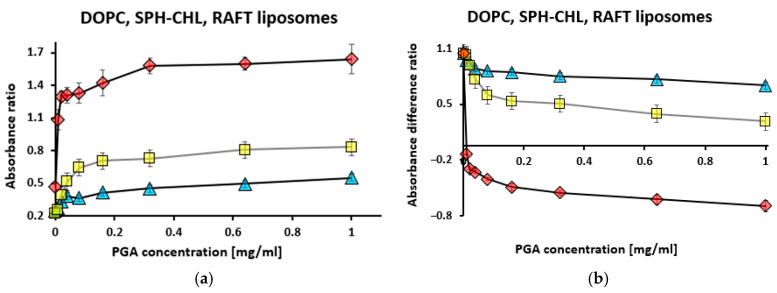
Absorbance ratio R_1_ = A_560_/A_630_ (**a**) for RAFT liposomes (red diamonds), DOPC liposomes (blue triangles), and SPH–CHL liposomes (yellow squares) along with absorbance difference ratio R_2_ (**b**) for RAFT liposomes, DOPC, and SPH–CHL liposomes during titration with polygalacturonic acid (PGA). Toluidine blue concentration was 10 µM, liposome concentration—0.4 mg/mL. R_2_ = (A_630_ – A_560_) (for samples with sequentially increasing concentrations)/(A_630_ – A_560_) (for the sample with the lowest concentration)). Values are the means ± SE of 3 independent experiments.

Based on these absorbance readings, the absorbance ratio (R_1_) and the absorbance difference ratio (R_2_) were calculated for RAFT and DOPC liposomes (Figure 5).

For RAFT liposomes, the absorbance ratio (R_1_) initially increased sharply from 0.47 to 1.30, followed by a gradual increase to 1.64 (Figure 5a). On the contrary, for DOPC and SPH–CHL liposomes, R_1_ slowly increased only to 0.38 at 0.04 mg/mL PGA and to 0.64 at 0.08 mg/mL PGA before increasing gradually to 0.55 and 0.83, respectively. This suggests that RAFT liposomes serve as more sensitive detectors for PGA at lower concentrations, while DOPC and SPH–CHL liposomes exhibit better detection at higher PGA concentrations. For RAFT liposomes, the absorbance difference ratio (R_2_) decreased sharply with an increasing PGA concentration before stabilizing at a negative value of 0.6 (Figure 5b), whereas for DOPC and SPH–CHL liposomes, the R_2_ value slowly decreased starting at 0.08 mg/mL PGA. During PSA titration, a similar stabilization of absorbance difference ratio R_2_ was observed (Figure 2b) in comparison with PGA titration (Figure 5b).

#### 3.2.3. Titration with Liposomes at Constant Toluidine Blue and Polygalacturonic Acid Concentrations

During titration with liposomes at concentrations ranging from 0 to 2 mg/mL, with fixed TB at 10 µM and PGA at 1 mg/mL, an increased absorbance was observed at 560 nm for both RAFT and DOPC liposomes (Supplementary Figure S6).

**Figure 6:**
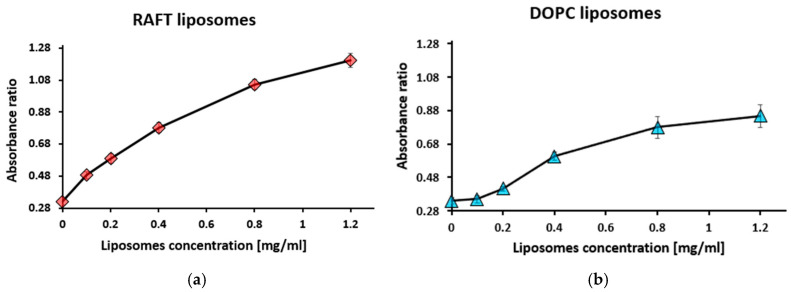
Absorbance ratio R_1_ = A_560_/A_630_ during titration with RAFT liposomes (**a**) and DOPC liposomes (**b**). Toluidine blue concentration was 10 µM, polygalacturonic acid concentration—1 mg/mL. Values are the means ± SE of 3 independent experiments.

Using these absorbance values, absorbance ratio R_1_ for RAFT and DOPC liposomes was determined (Figure 6).

For RAFT liposomes, the absorbance ratio (R_1_) gradually increased with an increasing liposome concentration (Figure 6a). A similar trend was observed for DOPC liposomes (Figure 6b). These relationships for absorbance ratio R_1_ during titration with liposomes in the presence of PSA (Figure 3) and in the presence of PGA (Figure 6) were similar.

## 4. Discussion

This report presents research on the formation of liposome–polysaccharide complexes based on the interactions between three types of liposomes, DOPC liposomes (which contain a lipid bilayer in the liquid-disordered (Ld) state), RAFT liposomes (which contain coexisting Ld/Lo domains within the lipid bilayer) and SPH–CHL liposomes (which contain a lipid bilayer in the liquid-ordered Lo) state) [43,44,45], and two selected anionic polysaccharides, polysialic acid (PSA) and polygalacturonic acid (PGA). The analysis was conducted using a toluidine blue (TB) probe and the absorption spectroscopy technique. The reported data show that upon binding of negatively charged PSA or PGA chains to the liposomal surface, the TB absorption maximum shifted from 630 nm to 560 nm for RAFT liposomes. On the contrary, neither DOPC nor SPH–CHL liposomes displayed such an absorption shift in the presence of PSA or PGA. Since RAFT liposomes, in contrast to DOPC liposomes and SPH–CHL liposomes, contain coexisting Ld/Lo domains, they experienced thickness mismatch-driven line tension at the Ld/Lo boundaries. The observed absorption shift depends on the binding of negatively charged polysaccharides to membranes; thus, we conclude that these boundaries facilitate binding of negatively charged polysaccharides to membranes. This conclusion is further supported by the membrane behavior of another polyanionic molecule—an ssRNA oligomer with the length of ca. 110 nts [46]. Atomic force microscopy images of a hybrid lipid bilayer surface incubated with RNA show large irregular RNA aggregates in the membranes that appear at the boundaries of flattened lipid patches [46]. This observation is similar to the fluorescent micrographs [46] where RNA tended to concentrate at points where the bilayer was bent or otherwise disturbed.

Thus, the observed metachromatic shift in TB absorption suggests that negatively charged PSA and PGA chains interact with the Ld/Lo boundaries within RAFT liposome membranes. These RAFT liposomes serve as models for lipid rafts found in biological membranes, which are structurally more ordered regions rich in sphingomyelin (SPH) and cholesterol (CHL). These lipids stabilize the bilayer, forming the Lo phase.

Two key parameters were utilized in the analysis: R_1_, which represents the absorbance ratio at 560 nm and 630 nm, and R_2_, which was employed for PSA and PGA titration by normalizing the absorbance difference at 630 nm and 560 nm relative to the baseline measurement at 0 mg/mL acid concentration. We chose these two simple parameters because of the potential applications of this system in the construction of nano-biosensors. Variations in absorbance at 560 nm and 630 nm across TB, liposome, and PSA/PGA titration experiments confirmed that PSA and PGA exhibited stronger binding to lipid raft-enriched membranes than to non-raft liposomes. The minimum PSA concentration required for detectable binding to liposomal membranes was 0.02 mg/mL, whereas the lowest liposome concentration facilitating PSA attachment was 0.4 mg/mL. TB was able to detect PSA attachment at concentrations as low as 3.74 µM. Similarly, the minimum PGA concentration for membrane binding was 0.01 mg/mL, with a required liposome concentration of 0.1 mg/mL. TB could detect PGA binding at concentrations as low as 4.42 µM. RAFT liposomes demonstrated greater sensitivity to PGA at low concentrations, whereas DOPC liposomes were more effective at detecting PGA interactions at higher concentrations.

Studies indicate that PSA binds more efficiently to positively charged liposomal membranes, such as those composed of ODA/DOPC (where ODA, octadecylamine, introduces a cationic charge due to an amine group), compared to neutral DOPC membranes [40]. However, PSA chains also exhibit enhanced affinity for electrically neutral lipid membranes containing ordered regions [47]. Therefore, there are at least two driving forces for PSA adsorption onto the membrane surface: the presence of positively charged lipids and the presence of lipid raft domains in the membranes. The TB monochromatic shift (from 630 nm to 560 nm) observed during titration of ODA/DOPC liposomes with PSA results from the presence of positively charged groups on the liposomal surface [40]. In contrast, the TB monochromatic shift during titration of RAFT liposomes with PSA results from the presence of lipid rafts (in the liquid-ordered state) within the liposomal membrane.

Research on neutrophil involvement in cancer metastasis has highlighted PSA’s ability to form stable nanocomplexes through electrostatic interactions with cationic drugs, making PSA-modified carriers promising for targeted drug delivery in metastatic breast cancer treatment [14]. PSA’s biocompatibility, biodegradability, and non-immunogenicity further enhance its potential for biomaterial applications, including the stabilization of proteins via polysialylation to extend their functional lifespan. Additionally, PSA has been explored in tissue engineering and as a key component in novel drug delivery platforms [13].

PGA, a linear polymer of galacturonic acid with exposed carboxyl groups, participates in electrostatic and hydrogen bonding interactions. Under physiological conditions, its negative charge facilitates interactions with positively charged liposomal surfaces [48]. Studies on gastrointestinal epithelial structures indicate that exogenous carbohydrates such as PGA exhibit bioadhesive and antimutagenic properties, suggesting a potential role in dietary interventions for cancer prevention and in treatments for inflammatory conditions such as Crohn’s disease and ulcerative colitis [35]. Stability studies of PGA–liposome complexes have demonstrated their resilience, with polydispersity indices ranging from 0.2 to 0.3 and zeta potential values ranging between −17 mV and −32 mV [19]. PGA–liposome formulations have been used for antimicrobial applications, with encapsulated lysozyme and nisin successfully inhibiting *Listeria monocytogenes* and *Salmonella enteritidis*, underscoring their potential for food safety applications [36].

The interaction between pectin variants and oppositely charged liposomes has also been explored. The principal chemical component of pectin is galacturonic acid. Pectin adsorption onto positively charged liposomes has been evidenced by increased particle size and a shift in zeta potential from positive to negative. However, no significant changes were observed when negatively charged liposomes were used, likely due to electrostatic repulsion. Given its mucoadhesive properties, pectin has been proposed as a stabilizing agent for liposome-based drug delivery systems [37]. It has been found that an anionic polysaccharide glypican-4 (a heparan sulfate proteoglycan) is localized to both lipid rafts and outside lipid rafts, and the localization affects its ability to regulate distinct Wnt pathways [49]. Other heparan sulfate proteoglycans syndecan-1 and syndecan-4 specifically associate with membrane raft microdomains, which could play a regulatory role in signal transduction [50].

Further studies on saccharic acids have reinforced the biomedical potential of sugar-based nanostructures. Liposomes coated with hyaluronic acid containing C12GEM demonstrated enhanced cytotoxic activity against pancreatic adenocarcinoma cell lines with CD44 overexpression [51]. Similarly, chondroitin sulfate-functionalized liposomes improved drug uptake by MDA-MB-231 breast cancer cells and showed promise as targeted drug carriers for solid tumors [52]. Chondroitin sulfate–liposome systems have also exhibited protective effects against oxidative damage, demonstrating superior anti-inflammatory activity compared to free chondroitin sulfate or non-functionalized liposomes [53]. Beyond biomedical applications, multilamellar vesicles (MLV) and nanoliposomes (SUV) derived from crude soy lecithin have been investigated for environmental remediation. SUV liposomes were shown to remove 85% of oil and aviation fuel contaminants from soil, with increased temperature further improving decontamination efficiency [2]. Encapsulated bacterial consortia with hydrocarbon degradation capabilities have also been explored, demonstrating rapid adaptation and achieving 90% pollutant removal over 165 days. This suggests that liposome-encapsulated bacteria and nutrients may enhance survival rates and biodegradation efficiency in hydrocarbon-contaminated environments [5]. In order to increase the stability of liposomes as nano-biosensors, polysaccharides (that mimic the cytoskeleton) are used [54]. In addition, multilayer polyelectrolytes (PEM) can be formed on the surface of liposomes, for example, from polysaccharides, and they are used in environmental protection to remove metal dyes and other pollutants [55].

TB has also been applied in photodynamic antibacterial studies. When conjugated with sophorolipid, TB exhibited enhanced bactericidal activity against *Pseudomonas aeruginosa* and *Staphylococcus aureus* [56]. Encapsulated TB within nanoliposomes demonstrated efficacy against *Streptococcus mutans*, a major contributor to dental caries [57], highlighting potential applications in oral healthcare. Additionally, TB has been used to assess cartilage degradation in osteoarthritis models, incorporated into injectable liposome-based hydrogels [58] containing teriparatide and gallic acid-grafted gelatin. Further studies have explored its role in reducing methemoglobin levels via electron mediation through glycolysis-derived red blood cell metabolites encapsulated in liposomes [59]. The change of spectroscopic properties of TB molecules by interaction with biological molecules has resulted in applications of TB in the field of sensor and nano-biosensor development [60].

## 5. Conclusions

This study evaluated PSA and PGA interactions with liposomes using absorption spectroscopy with TB. The results demonstrate that the presence of lipid raft domains, i.e., a coexistence of the Ld/Lo phases, significantly enhances the binding affinity of negatively charged polysaccharides to lipid bilayers. Additionally, TB proved to be a reliable indicator for detecting these interactions. These findings are important in light of the fact that liposome–polysaccharide complexes have potential applications in biomedicine (e.g., cancer therapy, antibacterial treatments), environmental remediation, water purification, and the development of nano-biosensors. Further research should explore additional polysaccharide–liposome interactions and optimize these complexes for practical applications.

## Data Availability

The original contributions presented in this study are included in the article/Supplementary Materials. Further inquiries can be directed to the corresponding author.

## Supplementary Materials

The following supporting information can be downloaded at: https://www.mdpi.com/article/10.3390/membranes15050139/s1, Figure S1: Absorption spectra of toluidine blue in the presence of 1 mg/mL polysialic acid and 0.5 mg/mL RAFT liposomes (a) or DOPC liposomes (b) during titration with toluidine blue; Figure S2: Absorption spectra of toluidine blue (TB) in the presence of 10 μM TB and 0.4 mg/mL RAFT liposomes (a), DOPC liposomes (b) or SPH-CHL liposomes (c) during titration with polysialic acid; Figure S3: Absorption spectra of toluidine blue (TB) in the presence of 10 μM TB and 1 mg/mL polysialic acid during titration with RAFT liposomes (a) or DOPC liposomes (b); Figure S4: Absorption spectra of toluidine blue for 1 mg/mL polygalacturonic acid and 0.5 mg/mL RAFT liposomes (a) or DOPC liposomes (b) during titration with toluidine blue; Figure S5: Absorption spectra of toluidine blue for 10 μM toluidine blue and 0.4 mg/mL RAFT liposomes (a), DOPC liposomes (b) or SPH-CHL liposomes (c) during titration with polygalacturonic acid; Figure S6: Absorption spectra of toluidine blue for 10 μM toluidine blue and 1 mg/mL polygalacturonic acid during titration with RAFT liposomes (a) or DOPC liposomes (b).
