# Supplementary material for: Toluidine Blue for the Determination of Binding of Anionic Polysaccharides to Lipid Raft Domains by Absorption Spectroscopy
Source: Membranes (Basel). 2025 May 2;15(5):139. doi: 10.3390/membranes15050139 (PMC12112939; doi:10.3390/membranes15050139)
Supplement: Supplementary file 1 [file membranes-15-00139-s001.zip › membranes-3529259-supplementary.pdf]

Toluidine Blue for Determination of Binding of Anionic Polysaccharides to Lipid Raft Domains by Absorption Spectroscopy

Sandra Gębczyńska, Julia Gdowska, Agata Mikos, Iga Gawrońska, Teresa Janas, Aleksander Czogalla and Tadeusz Janas*

Supplementary Material

Supplementary Figure S1.

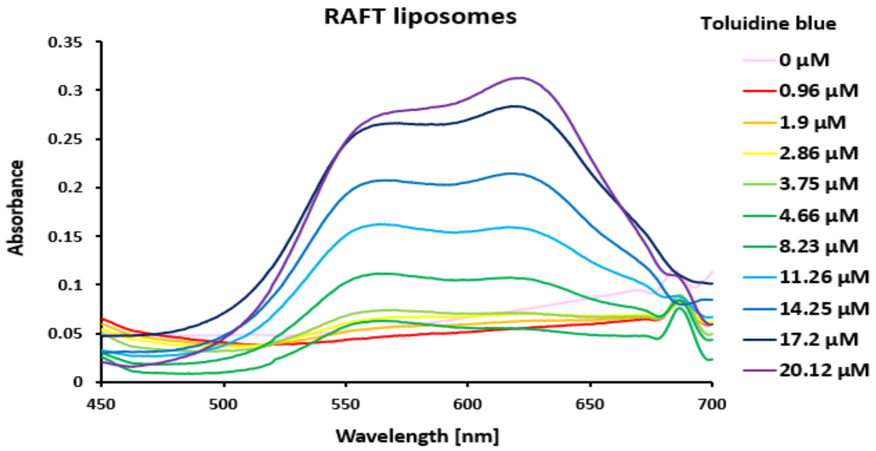

(a)

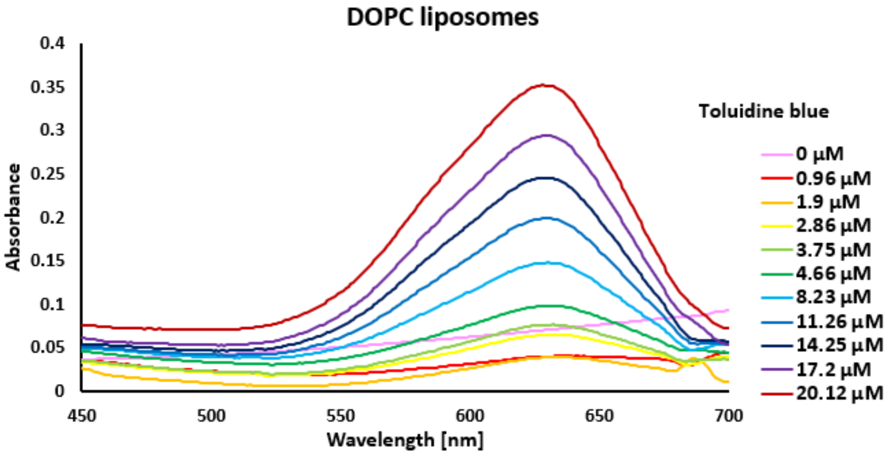

(b)

Figure S1. Absorption spectra of toluidine blue in the presence of 1 mg/ml polysialic acid and 0.5 mg/ml RAFT liposomes (a) or DOPC liposomes (b) during titration with toluidine blue.

Supplementary Figure S2.

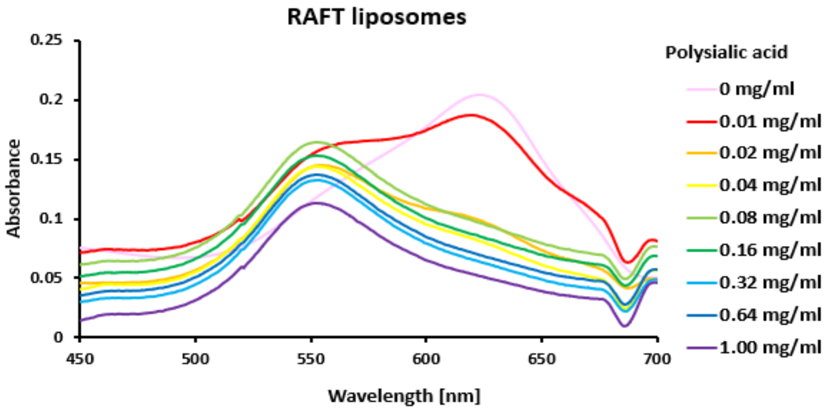

(a)

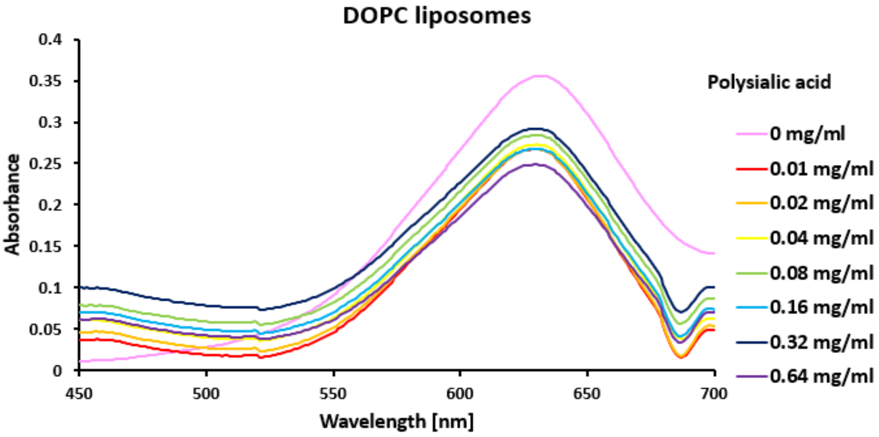

(b)

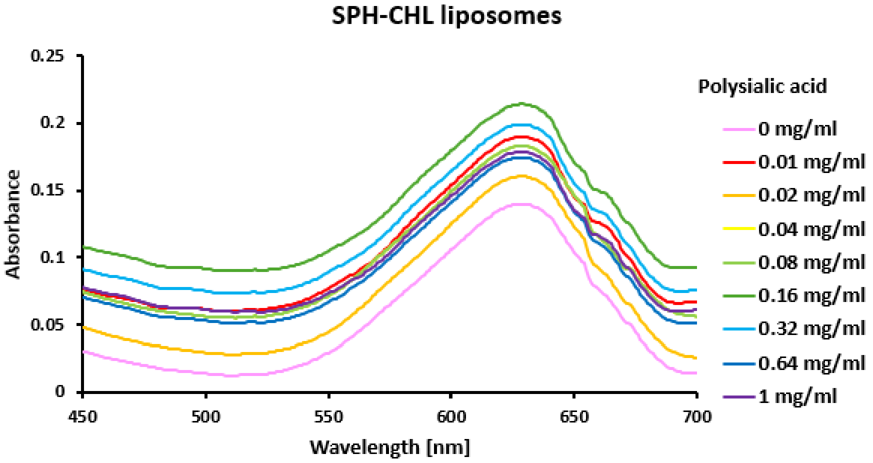

(c)

Figure S2. Absorption spectra of toluidine blue (TB) in the presence of 10 μM TB and 0.4 mg/mL RAFT liposomes (a), DOPC liposomes (b) or SPH-CHL liposomes (c) during titration with polysialic acid.

Supplementary Figure S3.

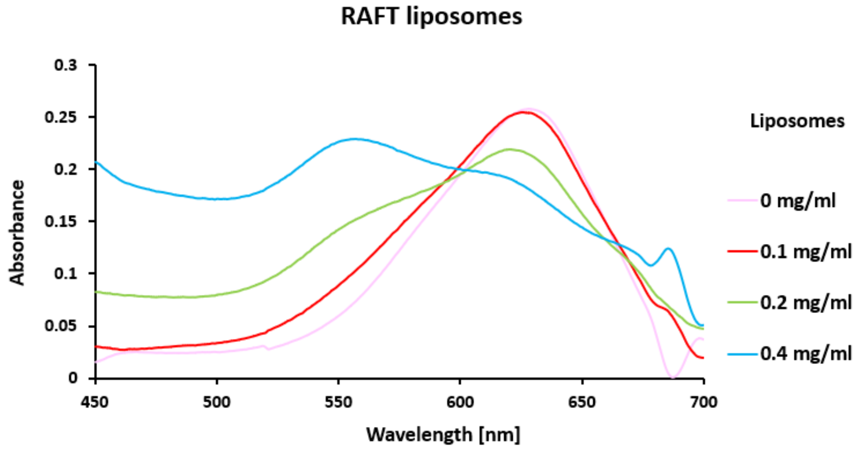

(a)

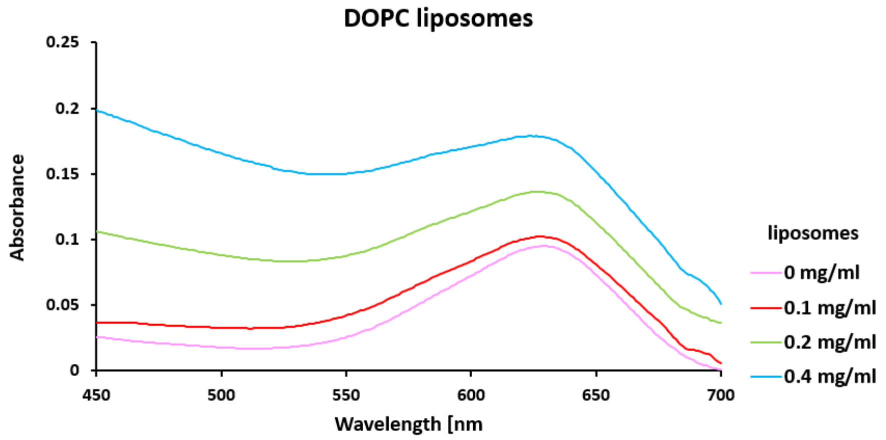

(b)

Figure S3. Absorption spectra of toluidine blue (TB) in the presence of 10 µM TB and 1 mg/ml polysialic acid during titration with RAFT liposomes (a) or DOPC liposomes (b).

Supplementary Figure S4.

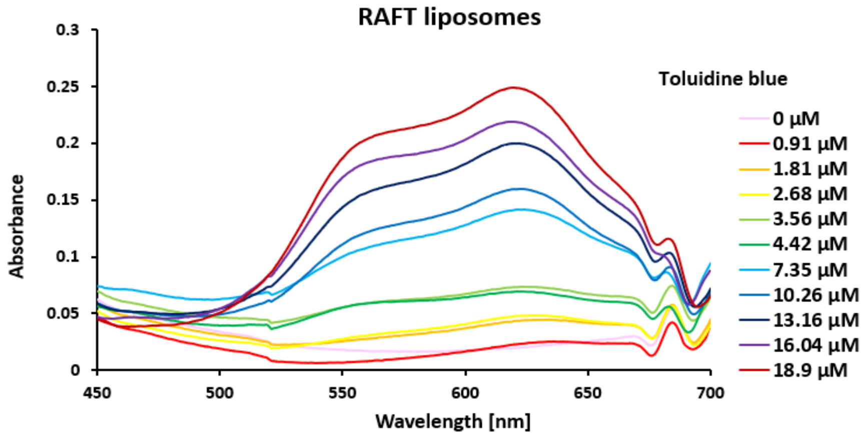

(a)

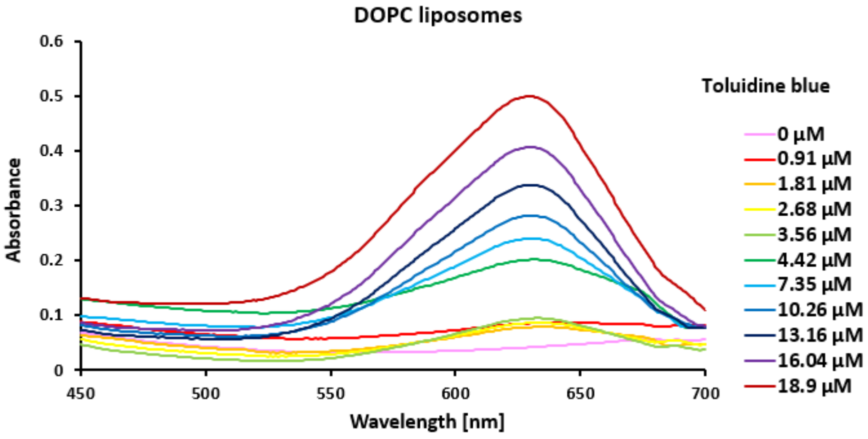

(b)

Figure S4. Absorption spectra of toluidine blue for 1 mg/ml polygalacturonic acid and 0.5 mg/ml RAFT liposomes (a) or DOPC liposomes (b) during titration with toluidine blue.

Supplementary Figure S5.

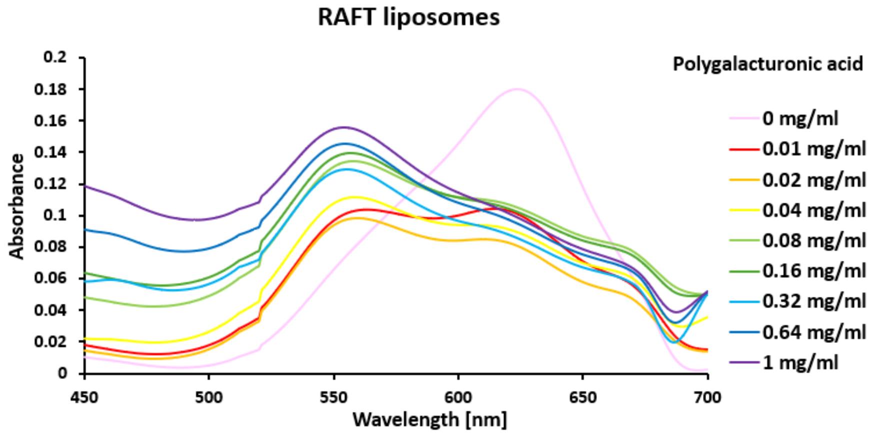

(a)

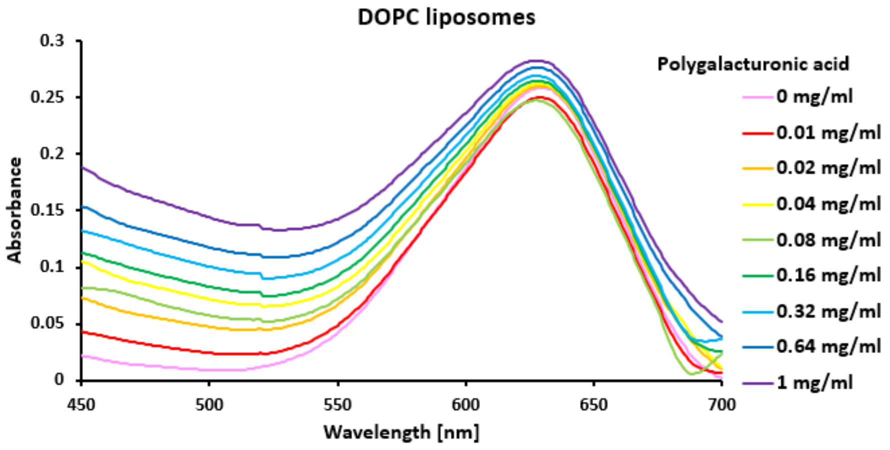

(b)

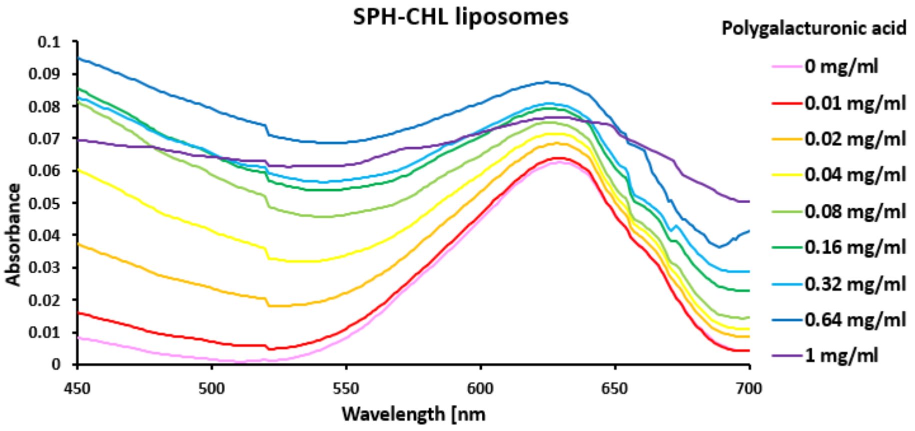

(c)

Figure S5. Absorption spectra of toluidine blue for 10 µM toluidine blue and 0.4 mg/mL RAFT liposomes (a), DOPC liposomes (b) or SPH-CHL liposomes (c) during titration with polygalacturonic acid.

Supplementary Figure S6.

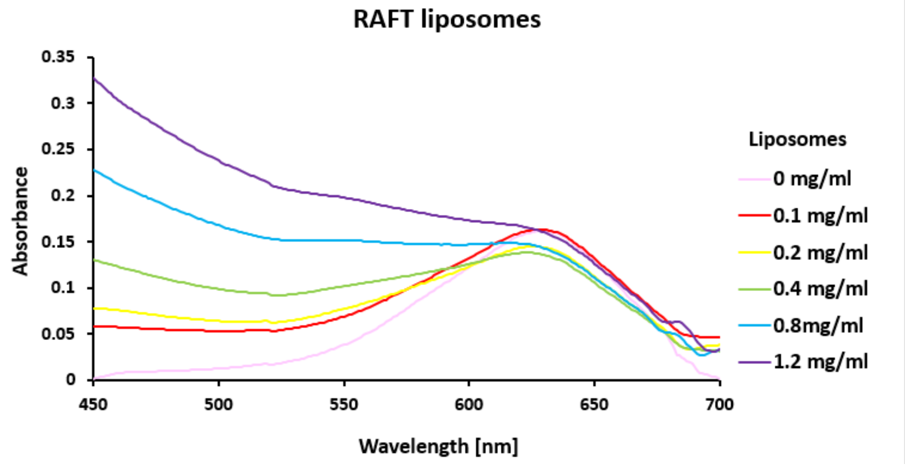

(a)

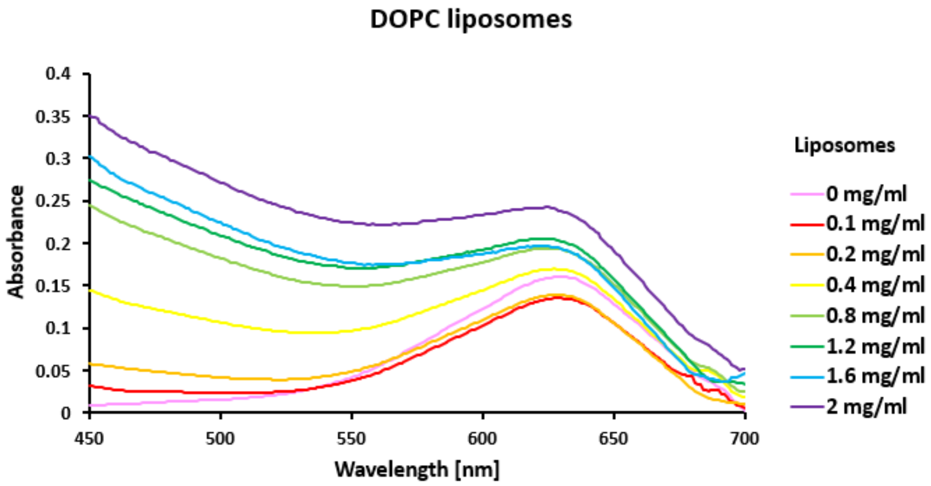

(b)

Figure S6. Absorption spectra of toluidine blue for 10 µM toluidine blue and 1 mg/ml polygalacturonic acid during titration with RAFT liposomes (a) or DOPC liposomes (b).
